# Global longitudinal strain for early detection of subclinical left ventricular systolic dysfunction in patients with systemic lupus erythematosus at a tertiary care centre: A hospital-based study

**DOI:** 10.6026/973206300220646

**Published:** 2026-02-28

**Authors:** Krati Mishra, Vinod Khandait, Kaustubh Telang, Suresh Sarwale, Dheeraj Karde, Saransh Barai

**Affiliations:** 1 Department of Medicine, Government Medical College & Hospital (GMCH), Nagpur, India; 2 Department of Rheumatology, SSH Nagpur, India; 3 Department of Cardiology, Super Speciality Hospital (SSH), Nagpur, India

**Keywords:** Systemic lupus erythematosus (SLE), global longitudinal strain (GLS), subclinical systolic dysfunction, echocardiography, speckle tracking, left ventricular function

## Abstract

Systemic Lupus Erythematosus (SLE) silently affects cardiac function, requiring early myocardial dysfunction detection to prevent morbidity. This hospital-based cross-sectional study assessed 49 SLE patients (2019 ACR/EULAR criteria) via clinical exam, Electrocardiogram (ECG) and echocardiography including speckle-tracking Global Longitudinal Strain (GLS). Evaluated parameters included Left Ventricular Ejection Fraction (LVEF), diastolic function, valvular lesions, pericardial effusion, pulmonary pressures and GLS (values < -16% indicating subclinical systolic dysfunction). Abnormal GLS occurred in 55.1%, with 28.5% showing preserved LVEF but impaired GLS, alongside systolic dysfunction (63.2%) and diastolic dysfunction (14.2%). GLS advances SLE cardiac screening by detecting subclinical LV impairment before LVEF decline, enabling timely intervention in preserved ejection fraction patients.

## Background:

Multiple organ systems are impacted by chronic autoimmune illness known as systemic lupus erythematosus (SLE) [1]. It occurs due to abnormal immune responses, where the body produces autoantibodies that often target nuclear antigens such as double- stranded DNA, histones and other cellular components. Immune complexes formed in circulation deposit in various tissues, leading to chronic inflammation and damage [1]. SLE's clinical manifestation varies greatly, involving the skin (malar rash, discoid rash, photosensitivity), joints (arthritis), kidneys (lupus nephritis), central nervous system (seizures, psychosis) and blood cells (anaemia, leukopenia, thrombocytopenia), which often occur in flares, alternating with periods of remission [1, 2]. Cardiovascular involvement is frequent but often silent, affecting the pericardium, myocardium, endocardium, coronary arteries and conduction system [3, 4]. Studies report that nearly 50% of SLE patients may develop cardiovascular disease (CVD) during the course of illness. SLE patients have a markedly higher risk of myocardial infarction, stroke and early atherosclerosis compared to the general population [5, 6-7]. Libman-Sacks endocarditis is a hallmark valvular lesion in SLE. It typically affects the mitral and aortic valves, leading to thickening, regurgitation, or stenosis [8]. Although these non-bacterial verrucous vegetations are frequently asymptomatic, they can raise the risk of infectious endocarditis and embolic events.

Patients with antiphospholipid antibodies are more likely to develop valve disease. Echocardiography serves as a non-invasive, easily accessible and cost-effective instrument for early identification of cardiac abnormalities in SLE patients. Advanced echocardiographic techniques, such as speckle-tracking and global longitudinal strain (GLS), can detect subtle myocardial changes even before symptoms develop or ejection fraction declines [9, 10]. Detecting subclinical abnormalities early provides an opportunity for timely intervention, close monitoring and risk modification. In the Indian context, especially in Central India, there is limited hospital-based data assessing the burden and spectrum of cardiac abnormalities in SLE patients using echocardiography. The absence of region-specific data limits clinical awareness and delays timely diagnosis and intervention. Regardless of whether cardiac symptoms are present, a comprehensive assessment of cardiac involvement in SLE patients is necessary to facilitate early detection and avoid consequences. Therefore, it is of interest to determine the prevalence of cardiac abnormalities detected by echocardiography in patients with Systemic Lupus Erythematosus.

## Methodology:

This hospital-based descriptive cross-sectional observational study was conducted in the Department of Medicine at the tertiary care centre in Central India. Ethical approval was obtained from the Institutional Ethics Committee. All research activities involving human participants followed ethical standards of the institutional research committee and the 2013 revised Declaration of Helsinki. The study was conducted from November 2022 to April 2025 by enrolling a total of 49 participants. Participants were recruited from a pool of SLE patients attending the outpatient department and those admitted to the medicine wards of a tertiary care hospital.

## Inclusion criteria:

SLE patients fulfilling 2019 ACR/EULAR criteria, providing informed consent.

## Exclusion criteria:

Patients with other rheumatologic diseases, known rheumatic heart disease or establish ischemic heart disease.

## Sampling:

A non-probability convenience sampling method was adopted. A total of 49 eligible patients were enrolled.

## Data collection procedure:

All patients attending the Medicine OPD or admitted to the Medicine wards of GMCH and SSH, Nagpur, were screened for eligibility based on the 2019 ACR/EULAR criteria for the diagnosis of SLE. The PI reviewed clinical records and laboratory data to assess whether patients met the inclusion and exclusion criteria. Only those who fulfilled all eligibility requirements were selected for participation. After confirming eligibility, the PI explained the nature and purpose of the study to each participant in their preferred language. Written informed consent was obtained using a bilingual consent form. Confidentiality and the ability to discontinue participation at any time without compromising their regular care were guaranteed to participants. Each enrolled participant was interviewed to gather information on sociodemographic details, duration of illness and relevant clinical features and recorded in a predesigned structured proforma. After obtaining informed consent, each participant underwent a detailed clinical examination. Laboratory investigations were performed to assess baseline clinical parameters and SLE-specific immune activity.

## Echocardiographic assessment:

All participants underwent transthoracic echocardiography (TTE) conducted by a trained cardiologist using a Philips Epic 7C echocardiography machine with a 2.5 MHz transducer.

## Global Longitudinal Strain (GLS):

GLS was assessed in all patients where technically feasible and image quality was adequate [11, 12-13]. GLS was measured using two-dimensional speckle tracking echocardiography (2D-STE). Standard apical views (apical four-chamber, two-chamber and long-axis views) were acquired and analysed offline on echocardiography machine (Philips Epic 7C) using dedicated software. The software automatically tracked speckles (acoustic markers) in the myocardium throughout the cardiac cycle to compute longitudinal strain. The GLS value was expressed as a negative percentage (%) because myocardial shortening during systole represents negative strain. A more negative value indicates better myocardial contractility. The following cut-off values were used: [14, 15] GLS > -18%: Normal global longitudinal strain, GLS between -16% to -18%: Borderline/indeterminate range and GLS < -16%: Abnormal strain, indicative of subclinical systolic dysfunction.

## Statistical analysis:

Continuous variables were compared using t-tests and expressed as mean ± standard deviation. Frequencies and percentages were used to represent categorical variables, which were then compared using either Fisher's exact test or the Chi-square test, depending on the situation. A p-value < 0.05 was considered statistically significant. All the statistical and graphical analysis for this study was undertaken by SPSS software version 26.0.

## Results:

The study included 49 patients diagnosed with Systemic Lupus Erythematosus (SLE). The majority of participants were young adults between 21 and 40 years, accounting for 67.4% of the total study population. Females constituted 89.8% of the cases, reflecting the known female predominance of SLE. Most patients (93.9%) were from urban areas. More than half (51%) had disease duration between 1 and 5 years, while 38.8% were newly diagnosed cases (Table 1). Echocardiographic parameters showed a mean left ventricular ejection fraction (LVEF) of 48.7 ± 13.7%, with a mean Global Longitudinal Strain (GLS) value of -15.4 ± 6.31%, suggesting subclinical systolic dysfunction in a substantial proportion. The mean E/A ratio was 1.32 ± 0.53 and the mean E/e' ratio was 8.26 ± 2.9, indicating that most patients had preserved diastolic function. The mean pulmonary artery systolic pressure (PASP) was 29.4 ± 9.85 mmHg (Table 2). Analysis of other echocardiographic findings revealed that 18.4% of patients had concentric left ventricular hypertrophy, while 6.1% showed global hypokinesia. A total of 65.3% had preserved LVEF (>50%), whereas 14.2% had LVEF below 40%. GLS assessment showed abnormal strain (<-16%) in 55.1% of patients, borderline values in 16.3% and normal strain (>-18%) in 28.5%. Notably, 28.5% of participants had normal LVEF but abnormal GLS, indicating subclinical systolic dysfunction. Diastolic dysfunction was found in 14.2% of cases, with 6.1% showing impaired relaxation and 6.1% showing restrictive filling pattern. Pulmonary hypertension and congenital lesions were rare findings (Table 3).

## Discussion:

In this hospital-based cross-sectional study, findings revealed a high prevalence of abnormal GLS in 55.1% of patients, with 28.5% exhibiting subclinical systolic dysfunction despite preserved left ventricular ejection fraction (LVEF). LV systolic dysfunction was observed in 63.2% of cases, diastolic dysfunction in 14.2% and other abnormalities, such as pericardial effusion and valvular lesions, were also noted. These results highlight the significant burden of subclinical cardiac involvement in SLE patients from this region and emphasise the value of GLS as a sensitive tool for early detection, potentially enabling preventive strategies to reduce cardiovascular complications. The demographic profile showed a strong female predominance (89.8%), which aligns with the typical presentation of SLE in other studies. This is supported by the Taha *et al.* study, with a female proportion of 89% [16] and Gegenava *et al.* also observed a predominantly female population [17]. In echocardiographic findings, the mean LVEF was 48.7%, which is lower than what is typically expected in SLE patients without overt cardiac disease. In comparison, Taha *et al.* reported a median LVEF of 60% in active SLE patients, while Zhang *et al.* found a mean LVEF of 60.6% in a largely asymptomatic group [16, 18]. These contrasts indicate that patients may have been at a more advanced disease stage or had more frequent myocardial involvement. Mohamed *et al.* reported a mean LVEF of 54%, which is slightly higher than in the present study, but they also documented subtle systolic impairment in a subset of patients [7]. Global longitudinal strain (GLS) provides a clearer picture of subtle dysfunction. The mean GLS was -15.4%, which is consistent with Gegenava *et al.* across all included studies in their meta-analysis [17]. Prior studies by Drissa *et al.* (-16.2%), Taha *et al.* (-19.9%) and Bourré-Tessier *et al.* support GLS utility in detecting subclinical dysfunction despite normal LVEF [16, 19 and 20]. Zhang *et al.* [18] also showed significantly lower strain across all myocardial layers, particularly in the endocardial strain, reinforcing that SLE affects contractility early, aligning with the current findings. In the present study, more than half of the patients (55.1%) had abnormal global longitudinal strain (GLS) values below 16%, while 16.3% had borderline values between 16% and 18%. Only 28.5% showed normal GLS above 18%; these are consistent findings across studies. Drissa *et al.* [19] reported abnormal GLS in 52%, Bourré-Tessier *et al.* [20] found reduced GLS in 48% and Mohamed *et al.* [7] also documented subtle systolic impairment using GLS. These findings across studies highlight GLS as a sensitive marker for subclinical ventricular dysfunction in this patient population. In the present study, 28.5% of patients had a normal left ventricular ejection fraction (LVEF) but abnormal global longitudinal strain (GLS). This finding highlights the presence of subclinical myocardial dysfunction that would not be identified using conventional ejection fraction assessment alone. The importance of GLS in SLE lies in its ability to detect these early myocardial changes, allowing clinicians to intervene before irreversible damage occurs. Bourré-Tessier *et al.* reported that reduced GLS was present in 48% of SLE patients with normal LVEF, supporting the present observation [20]. Drissa *et al.* similarly found abnormal GLS in 52% of cases, many with preserved LVEF, confirming that strain imaging can identify myocardial impairment at a stage when standard measures appear normal [19]. Mohamed *et al.* also demonstrated subtle myocardial dysfunction with GLS in patients who's LVEF remained within normal limits [7]. Additional evidence from recent literature further supports the role of myocardial deformation imaging in identifying subclinical ventricular dysfunction. Although not specific to SLE, Noori and Barzani demonstrated that GLS can detect early systolic dysfunction in patients with preserved LVEF, with impaired GLS closely related to increase left ventricular mass, myocardial remodelling and reduced MAPSE [21]. These findings emphasise the sensitivity of GLS as a marker of early myocardial impairment and support its broader applicability in systemic conditions associated with myocardial stress, including autoimmune diseases such as SLE. Recent studies have highlighted that cardiovascular involvement in SLE is frequent, often asymptomatic and multifactorial, driven by both traditional cardiovascular risk factors and disease-specific mechanisms such as inflammation, immune-mediated myocardial injury and antiphospholipid antibodies [22]. Contemporary echocardiography incorporating advanced techniques such as GLS has been recommended as an essential tool for early detection of myocardial involvement, enabling timely intervention before the development of overt cardiovascular disease [22]. Meta-analysis evidence also supports these observations. Di Minno *et al.* in a systematic review and meta-analysis reported that SLE patients had significantly lower left and right ventricular strain parameters compared with non-SLE controls, confirming the presence of global myocardial dysfunction even in the absence of overt cardiac disease [23]. Their analysis further indicated that female predominance and coexisting hypertension influenced GLS values, highlighting the complex interplay between disease-specific and conventional cardiovascular risk factors. Beyond GLS, newer strain-derived indices provide additional insight into myocardial mechanics in SLE. Zhong *et al.* demonstrated that noninvasive myocardial work indices derived from left ventricular pressure strain loops, particularly global work efficiency (GWE), were significantly reduced in SLE patients compared to controls, with more pronounced abnormalities in those with lupus nephritis. GWE outperformed GLS in detecting early myocardial dysfunction [24]. Collectively, these findings reinforce the role of GLS as a sensitive, non-invasive marker for detecting early cardiac involvement in SLE, which is crucial for guiding timely therapeutic strategies and potentially improving long-term cardiovascular outcomes in this patient population.

## Conclusion:

We show a high prevalence of subclinical left ventricular systolic dysfunction in SLE patients from Central India, detected primarily through abnormal GLS despite preserved LVEF in many cases. As a sensitive, non-invasive method for early detection of myocardial involvement, GLS presents chances for prompt therapies to reduce cardiovascular risks. Routine integration of GLS into echocardiographic assessments is recommended to enhance cardiac care in this vulnerable population.

## Figures and Tables

**Table 1:** Characteristics of participants

**Variable**	**Category**	**N**	**%**
Age of Patients (years)	20-Nov	13	26.5
	21-30	16	32.7
	31-40	17	34.7
	41-50	3	6.12
Gender	Female	44	89.8
	Male	5	10.2
Residence	Rural	3	6.12
	Urban	46	93.9
Duration of SLE	Newly Diagnosed	19	38.8
	< 1 Year	2	4.08
	1-5 Years	25	51
	6-10 Years	2	4.08
	11-15 Years	1	2.04

**Table 2:** Echocardiographic parameters

**Variable**	**Mean**	**SD**
Left Ventricular Ejection Fraction	48.7	13.7
E/A Ratio	1.32	0.536
E/e' Ratio	8.26	2.9
Pulmonary Artery Systolic Pressure	29.4	9.85
Global Longitudinal Strain	-15.4	6.31
Left Ventricular Internal Diameter in Diastole	41.3	4.51
Left Ventricular Internal Diameter in Systole	29.9	4.46
Aorta	19.9	3.79
Left Atrium	31.3	11
Interventricular Septum	10.4	3.71
Posterior Wall	10	2.47

**Table 3:** Other echocardiography findings

**Variable**	**Category**	**N**	**%**
Left ventricular chambers	Concentric LVH	9	18.4
	Global LV hypokinesia	3	6.12
	Normal	37	75.5
LVEF	LVEF >50%	32	65.3
	40-50%	10	20.4
	<40%	7	14.2
GLS	>18%(Normal).	14	28.5
	16 to 18%(borderline)	8	16.3
	< 16% (Abnormal).	27	55.1
	Normal LVEF/Abnormal GLS	14	28.5
	Clot/Vegetation	0	0
	Congenital heart disease	1	2.04
E/A	<0.8 (impaired)	4	8.1
	0.8-1(borderline)	3	6.1
	1-2(normal)	39	79.5
	>2 (Restrictive).	3	6.1.
E/e'	<8 normal.	29	59.1
	8-14 borderline	17	34.6
	>14 impaired	3	6.1
LV Function	LV Systolic dysfunction	31	63.2
	LV Diastolic dysfunction	7	14.2
